# Neuronal metabolism role in ketogenic diet improvements in bortezomib-induced painful peripheral neuropathy mouse model

**DOI:** 10.1097/PR9.0000000000001414

**Published:** 2026-02-17

**Authors:** Lana L. Heslop, Trent K. Madden, Gentry Totta-Griese, Sarah J. Crowards, Will Hauser, Janelle Ryals, Kyle M. Baumbauer, Heather M. Wilkins, Douglas E. Wright

**Affiliations:** Departments of a Anesthesiology, Pain, and Perioperative Care; b Cell Biology, Physiology and; c Neurology, University of Kansas Medical Center, Kansas City, KS, USA

**Keywords:** Ketogenic diet, Bortezomib, Ketones, Neurites, Sensory neurons, Neuropathy

## Abstract

**Introduction:**

A ketogenic diet (KD) can benefit a range of neurological conditions, including different types of peripheral neuropathy. Bortezomib (BTZ), a first-line chemotherapy for treating multiple myeloma, has neurotoxic effects and often induces chemotherapy-induced peripheral neuropathy.

**Objectives:**

Here, we tested whether a KD could prevent the onset of small fiber neuropathy and behavioral hypersensitivity associated with bortezomib-induced peripheral neuropathy (BIPN).

**Methods:**

Male C57BL/6 mice were fed either a standard chow diet or a KD for 10 days. During the first 5 days of KD administration, mice received daily intraperitoneal injections of BTZ or vehicle. Mice entered a BTZ washout period on day 6 while remaining on their specific diets. Hindpaw responses to mechanical (von Frey) and cold (acetone) stimuli were measured to determine how KD and BTZ treatment affected behavioral sensitivity. Footpads were harvested for analysis of intraepidermal nerve fiber density (IENFD), and lumbar dorsal root ganglia were collected for in vitro analysis of neurite outgrowth and cellular bioenergetics.

**Results:**

Bortezomib-treated, KD-fed mice exhibited decreased mechanical hypersensitivity, normalized cold hypersensitivity, preserved IENFD, and decreased extracellular acidification rate compared with BTZ-treated, chow-fed mice. In vitro analysis of ketones' ability to protect against BTZ-induced reductions in neurite growth revealed that cultured neurons pretreated with ketones were protected from BTZ-induced neurite degeneration.

**Conclusion:**

Findings suggest that KD protects against reductions in IENFD, behavioral hypersensitivity, and bioenergetic shifts induced by BIPN, and provides evidence that KD provides protection against BTZ and could be a potential therapeutic for BIPN.

## Abstract 2

A ketogenic diet can mitigate bortezomib-induced painful peripheral neuropathy symptoms through altering the bioenergetic activity of sensory neurons.

## 1. Introduction

Bortezomib is a first-line chemotherapy drug used mainly for the treatment of multiple myeloma. Approximately 30% to 70% of patients with cancer who receive bortezomib (BTZ) develop bortezomib-induced peripheral neuropathy (BIPN).^1,2,40,46,47^ Bortezomib-induced peripheral neuropathy symptoms include pain, numbness, paresthesia, and altered thermal sensitivity in a “stocking and glove distribution.”^40,46,47^ Assessments of epidermal innervation suggest that patients with BIPN have reduced intraepidermal nerve fiber density (IENFD), consistent with a small-fiber neuropathy.^4,19^ Currently, there are no successful treatment options for BIPN. However, duloxetine, gabapentin, tricyclic antidepressants, and topical creams are commonly prescribed to try to reduce symptomatic neuropathic pain.^47^ The consequences of BIPN often lead to dose reduction or cessation of BTZ treatment, which both negatively affect patient quality of life and survival rates.^46,47^ Although BIPN symptoms may resolve over time, symptoms can also be chronic and often irreversible.^8,47^

Bortezomib is known to be mitotoxic and can increase glycolysis in DRG neurons, both of which contribute to the pathophysiology of BIPN.^34,47,48^ The maintenance of neuronal metabolism and bioenergetics represents a finely regulated balance, where dysregulation can have detrimental effects on cellular processes.^18,20^ Various studies have shown that abnormal glycolysis plays a role in the development of pain,^12,28,31,34,44^ while glycolytic inhibition alleviates pain.^23,32^ This highlights the role of metabolism and neuronal bioenergetics in pain, specifically in relation to glycolytic activity and signaling. Currently, there is little research regarding the effects of neuronal bioenergetics on axon degeneration and IENF density in peripheral neuropathy models.

Interest has grown in the role of ketones in pain modulation.^22^ Circulating ketones are elevated when consuming a ketogenic diet (KD), and evidence has revealed that ketones alter neuronal metabolism by decreasing reactive oxygen species production, increasing oxidative phosphorylation, and modulating nicotinamide adenine dinucleotide levels.^9,15,16,36^ A KD has numerous benefits for various neurological conditions and neurodegenerative diseases,^13,21,24,26,27,30,41,43^ leading us to test whether a KD can be used as an adjunct therapy in BIPN. Previous work from our laboratory has shown that a KD can prevent and rescue fiber loss and mechanical allodynia in several mouse models of metabolic-driven peripheral neuropathy (DPN).^17^ We believe that the benefits of a KD can be extended beyond DPN also to benefit BIPN. Our study aimed to explore the interplay among BTZ, ketones, and neuronal metabolism in peripheral neuropathy. Our data suggest that in mice, KD consumption prevents or normalizes key changes in behavior and sensory neuron metabolism induced by BTZ. These results extend the range of benefits for treating neuropathy and may provide new avenues for treating BIPN that involve neuronal bioenergetics.

## 2. Materials and methods

### 2.1. Animals and diets

All animal work was conducted following review and approval by the Institutional Animal Care and Use Committee of the University of Kansas Medical Center. Eight-week-old C57/Bl6 #027 male mice were purchased from Charles River Laboratories and maintained on a 14:10 light:dark cycle in the animal research facility at the University of Kansas Medical Center. Mice were given ad libitum access to water and either a control rodent chow (TD.8604, Envigo, Madison, WI; 14% fat, 32% protein, and 54% carbohydrate by kcal) or a ketogenic diet (TD.96355; Envigo, 90.5% fat, 9.2% protein, and 0.3% carbohydrate by kcal). Mice fed a ketogenic diet were given fresh ketogenic food every 3 to 4 days. After baseline behavior measures, mice were randomized into 1 of 4 groups: bortezomib or vehicle-treated, and receiving either a standard chow diet or a ketogenic diet. The diet was initiated at least 7 hours before the first injection. Mice were given an intraperitoneal injection of either BTZ (0.2 mg/kg) or vehicle for 5 consecutive days. Afterward, the mice were monitored daily for 5 days during a washout period.

### 2.2. Drug administration

For in vivo studies, bortezomib (Tocris, Catalog No. 7282) was dissolved in 0.5% DMSO (Fisher Scientific), then diluted in 0.9% saline (Hospira, Lake Forest, IL), and administered through intraperitoneal (i.p.) injection using a 1-mL insulin syringe with a 28 G needle (BD; Catalog No. 329420). Vehicle solution was 0.9% saline (Hospira; Lake Forest, IL) with 0.5% DMSO. Mice received an i.p. injection per day of either vehicle or BTZ (0.2 mg/kg) for 5 consecutive days. For in vitro studies, BTZ (100 nM) was dissolved in 0.5% DMSO, then diluted into DMEM (Gibco; Catalog No.11885-084).

### 2.3. Blood measurements

Blood ketone levels were measured once using a hand-held monitor and B-hydroxybutyrate blood ketone strips (β-Ketone blood test strips, Abbott Laboratories, Chicago, IL; Precision Xtra, Abbott Laboratories). Animals were not fasted before blood ketone measurements.

### 2.4. Mechanical sensitivity (monofilament) testing

Mice were placed in 11 cm diameter × 19 cm height cylindrical plexiglass containers on top of a 9.4 × 10-cm wire platform and allowed to acclimate to the testing environment for 30 minutes. Evoked mechanical sensitivity was assessed using nylon monofilaments of graded bending forces applied using the up-down method to the plantar surface of each hind paw. Testing was conducted in duplicate, with a minimum of 5 minutes between tests, to assess the average force that evokes a withdrawal response.

### 2.5. Cold sensitivity (acetone evaporation) testing

Fifteen minutes after mechanical sensitivity testing, cold sensitivity was assessed using acetone (Fisher Scientific). In brief, a droplet of acetone was placed on the plantar surface of 1 hind paw using a 1-mL insulin syringe with a 28 G needle (BD; Catalog No. 329420). Training was conducted before experiments to ensure that the acetone droplet could be placed on the footpad without touching the needle to the hind paw. The number of behavioral responses to the acetone-treated foot was recorded for 1 minute. Responses included paw shaking, licking, biting, favoring, or attending. Assessment of sensitivity was performed over the course of 2 trials on each hind paw. Response numbers were averaged for each paw, statistically compared to ensure no difference between paws, and averaged for final analysis and presentation.

### 2.6. Intraepidermal nerve fiber density

Footpads were collected from all mice and postfixed in Zamboni fixative for 18 hours before incubation in 30% sucrose overnight. Footpads were cryopreserved in Optimal Cutting Temperature Compound (Sakura Tissue-Tek) and cut into 30-μm sections. Footpad sections were blocked for 4 hours in Superblock (ThermoFisher, Grand Island, NY), 1.5% Normal Donkey Serum, 0.5% Triton X-100 (Sigma), and 0.5% Porcine Gelatin at room temperature. Slides were incubated overnight with rabbit a-PGP9.5 (1:1000, UCHL1; ProteinTech; Rosemont, IL). Slides were incubated for 1 hour with 555-tagged donkey-a-rabbit secondary antibody (1:1000; Alexa Fluor). Stained slides were imaged using a Nikon Eclipse 90i microscope with a ×20 objective. Three images were captured per tissue section, for a total of 9 images per slide. Nikon Elements was used to measure the length of the dermis–epidermis junction. Intraepidermal nerve fiber density was quantified as the number of fibers crossing the junction per millimeter. The average IENFD for each mouse was used for statistical analyses.

### 2.7. Neurite outgrowth

Methods were based on Malin et al.^38^ and modified as follows. Lumbar DRG neurons were harvested and dissociated into a single cell suspension. Cells were plated using the drop-plate method, in which a 40-μL drop is placed in the center of each well. Cells were incubated at room temperature for 1 to 2 hours and then fed with DMEM (Gibco; Catalog No. 11885-084) before being placed in a 37°C incubator. For in vivo studies, neurons were grown for 4 days and then fixed with 4% paraformaldehyde for 15 minutes. For in vitro studies, depending on the group, neurons received ketones (3 mM beta-hydroxybutyrate; Sigma-Aldrich and 3 mM acetoacetate; Sigma-Aldrich) 48 hours postplating and BTZ 96 hours postplating. Cells were fixed 144 hours postplating with 4% paraformaldehyde for 15 minutes. Immunocytochemistry was performed using Beta-III tubulin (1:1000; TUJ1; R&D Systems), Hoechst (1:2000; Invitrogen; Eugene, OR), and 488-tagged donkey-a-mouse secondary (1:2000, Alexa Fluor) stains. Plates were imaged, with 5 to 6 photographs per well and 3 to 4 wells per animal. Neurite outgrowth was quantified using ImageJ. A grid was superimposed on the well images. The number of somas producing neurites was counted, along with the number of square neurons (attached to a soma) that passed through. The average square-to-soma ratio and the total number of squares were calculated by averaging the results from each well and then averaging the well replicates to obtain a single standard value per animal.

### 2.8. Extracellular acidification rate and mitochondrial oxygen consumption rate

Lumbar DRG neurons were harvested and dissociated into a single cell suspension. Cells were plated in a poly-d-lysine–coated 96-well plate at 100 μL/well and fed with an additional 100 μL of media (either standard media or ketone media with 3 mM beta-hydroxybutyrate and 3 mm acetoacetate) after 2 hours. The following day, cells were analyzed for extracellular acidification rate and oxygen consumption rate using a Seahorse XFe96 analyzer (Agilent Technologies). Unbuffered DMEM containing 4 mM glutamine, pyruvate, and 5 mM glucose was added to plated cells, and ketones (as described previously) were added to the medium for the respective KD groups. The following substrates were injected in succession to measure ECAR and OCR: (1) 2 µM Oligomycin/Hoechst; (2) 0.5 µM Carbonyl Cyanide‐p‐trifluoromethoxyphenylhydrazone (FCCP); (3) 1 µM each Rotenone/Antimycin A; (4) 10 mM 2DG. Cells were counted for Hoechst stain in a Cytation 1 plate reader to normalize measures to cell counts.

### 2.9. Statistics and data analysis

All statistical analyses were performed using GraphPad Prism 10.5. All data are presented as mean ± standard error of the mean. All data collected over time were analyzed using a 2-way analysis of variance (ANOVA) with repeated measures. All other analyses were performed using a 1-way or 2-way ANOVA, or a Pearson correlation, as appropriate. Data were analyzed post hoc by Tukey Honest Significant Difference or pairwise *t* test, as indicated. Assumptions of normal distribution and homogeneity of variance were confirmed by the Shapiro–Wilk test.

## 3. Results

### 3.1. Mouse responses to bortezomib and ketogenic diet

**Figure 1.**
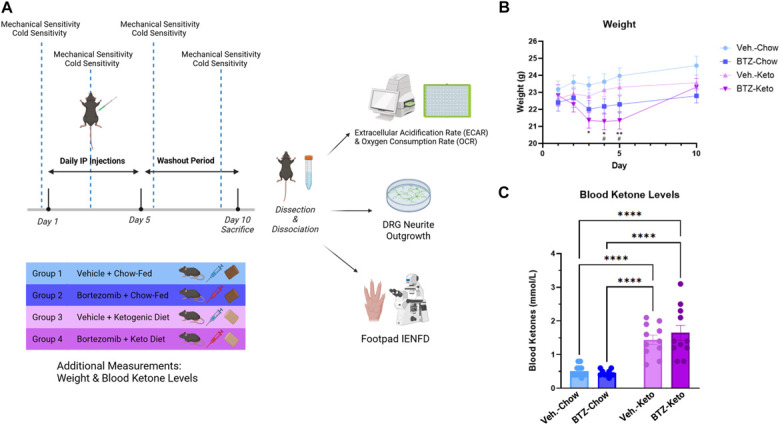
Depiction of the timeline and experimental design (A), weight over time (*P < 0.05 BTZ-Keto vs Veh.-Chow; #*P* < 0.05 BTZ-Keto vs Veh.-Keto) (B), and blood ketone levels (C), n = 11 to 12. BTZ, bortezomib; IENFD, intraepidermal nerve fiber density.

Ketogenic diet was administered to respective groups on day 1 (Fig. 1A). Vehicle-treated KD-fed mice did not have significant weight loss over the course of the experiment (Fig. 1B). The weight of BTZ-treated KD-fed mice was significantly different compared with vehicle-treated chow-fed animals on days 3 to 5, and significantly different from vehicle-treated KD-fed animals on days 4 and 5 (Fig. 1B, 2-way ANOVA with repeated measures, time: *P* < 0.0001, group × time: *P* < 0.0001). However, all mice were still within a healthy weight range for their age, sex, and strain. A blood sample for beta-hydroxybutyrate was collected on day 10 to confirm that KD-fed animals had entered ketosis. KD-fed animals had significant increases in blood ketone levels (Fig. 1C, 2-way ANOVA, diet: *P* < 0.0001).

### 3.2. Ketogenic diet mitigates bortezomib-induced peripheral neuropathy symptomology

**Figure 2.**
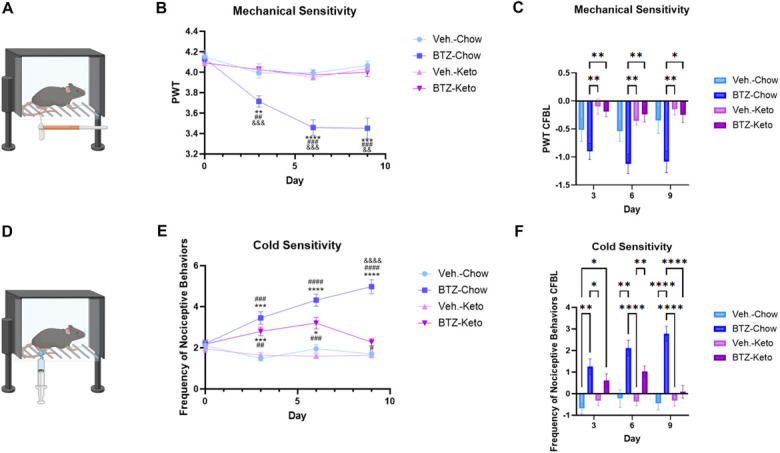
Illustration of the sensory behavior assays of mechanical sensitivity and cold sensitivity. Graphic depicts mechanical sensitivity protocol (A). Paw withdrawal threshold for mechanical sensitivity (*P < 0.05 BTZ-Chow vs Veh.-Chow; #*P* < 0.05 BTZ-Chow vs Veh.-Keto; &*P* < 0.05 BTZ-Chow vs BTZ-Keto) (B) and change from baseline of PWT (C) were measured. Graphic depicts cold sensitivity protocol (D) along with frequency of nociceptive behaviors (*P < 0.05 compared with Veh.-Chow; #*P* < 0.05 compared with Veh.-Keto) (E) and change from baseline of cold sensitivity (F), n = 11–12. BTZ, bortezomib; PWT, paw withdrawal threshold.

Baseline sensory behavioral testing (mechanical sensitivity and cold sensitivity) was measured on day 0 (Fig. 1A). Bortezomib treatment and KD administration began on day 1. Figure 2A illustrates mechanical sensitivity testing using von Frey nylon monofilaments. Bortezomib-treated chow-fed mice developed mechanical allodynia by day 3, which continued for the remainder of the experiment (Fig. 2B; 2-way ANOVA with repeated measures, group: *P* < 0.0001, group × time: *P* < 0.0001, time: *P* < 0.0001). A KD prevented the development of mechanical allodynia in BTZ-treated mice, maintaining mechanical thresholds similar to vehicle-treated, chow-fed controls. Figure 2C depicts the change from baseline, further highlighting differences in mechanical sensitivity thresholds between groups (2-way ANOVA with repeated measures; group: *P* = 0.0005).

Assessment of cold sensitivity revealed no difference between the vehicle-treated chow-fed group compared with the vehicle-treated KD-fed group (Fig. 2D, E, 2-way ANOVA with repeated measures, group: *P* < 0.0001, group × time: *P* < 0.0001, time: *P* < 0.0001). Bortezomib-treated groups had a significant increase in frequency of nociceptive behaviors (biting, licking, and favoring) when compared with vehicle-treated groups on days 3 and 6 (Fig. 2E, 2-way ANOVA with repeated measures, group: *P* < 0.0001, group × time: *P* < 0.0001, time: *P* < 0.0001). On day 9, BTZ-treated chow-fed mice showed an increase in the frequency of nociceptive responses. They remained significantly different from vehicle-treated groups (Fig. 2E). There were also fewer responses in the BTZ-treated KD-fed group on day 9 during the washout period and were not different from the vehicle-treated chow group but were different from vehicle-treated KD-fed mice (Fig. 2E). Figure 2F depicts the change from baseline, revealing that BTZ-treated chow-fed animals had a significant change from their baseline behaviors, along with BTZ-treated KD-fed animals (2-way ANOVA with repeated measures, group: *P* < 0.0001, group × time: *P* < 0.0001, time: *P* = 0.0082). However, behavioral changes in the BTZ-treated KD-fed mice were normalized by day 9.

### 3.3. Ketogenic diet protects against the development of bortezomib-induced peripheral neuropathy

**Figure 3.**
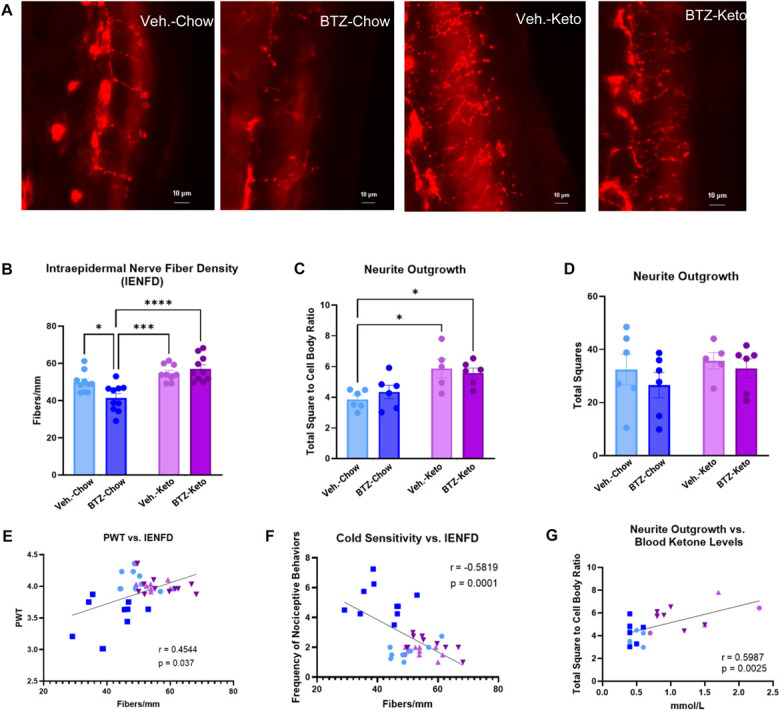
(A) shows representative images of IENFD from all mouse groups. Scale bars = 10 µm. (B) depicts quantification of IENFD results (n = 9–10). (C and D) illustrate measures of neurite outgrowth from DRG harvesting and culturing (n = 5–6 mice, 3–6 replicates). (E–G) represent correlations between IENFD, sensory behavior, blood ketone levels, and DRG neurite outgrowth. BTZ, bortezomib; DRG, dorsal root ganglia; IENFD, intraepidermal nerve fiber density.

Because BIPN causes fiber loss in patients^47^ and rodents,^6^ we tested whether a KD affects BIPN-induced reductions in IENFD (Fig. 3A). Bortezomib-treated chow-fed animals had a significant decrease in IENFD when compared with all groups (Fig. 3B, 2-way ANOVA, diet: *P* < 0.0001, drug × diet interaction: *P* < 0.0115). Vehicle-treated KD-fed animals had a significant increase in total square to cell body ratio from the harvested DRG compared with both chow-fed groups (Fig. 3C, D, 2-way ANOVA, diet: *P* < 0.0008). Bortezomib-treated KD-fed animals also had a significant increase in total square to cell body ratio when compared with vehicle-treated chow-fed animals. However, when comparing total squares alone, there was no significant difference between groups (Fig. 3D, 2-way ANOVA, *P* < 0.05).

We conducted correlations between the levels of IENFD and mechanical sensitivity (Fig. 3E), and IENFD and cold sensitivity (Fig. 3F). The results identified a significant correlation between IENFD and mechanical sensitivity (r = 0.4544, *P* < 0.0037) and cold sensitivity (r = −0.5819, *P* < 0.0001), suggesting that reduced IENFD correlates with a worse behavioral change. Finally, we tested whether there is a relationship between in vivo blood ketone levels and in vitro DRG neurite outgrowth (Fig. 3G). Results showed a significant correlation between the level of circulating ketones in vivo and neurite outgrowth of DRG in vitro (r = 0.5987, *P* < 0.0025). This revealed that ketone levels can influence features of DRG axons, even when cultured in vitro, suggesting that DRG neurons exhibit a metabolic memory in response to elevated ketone levels.

### 3.4. Bortezomib-induced metabolic alterations countered by ketogenic diet

**Figure 4.**
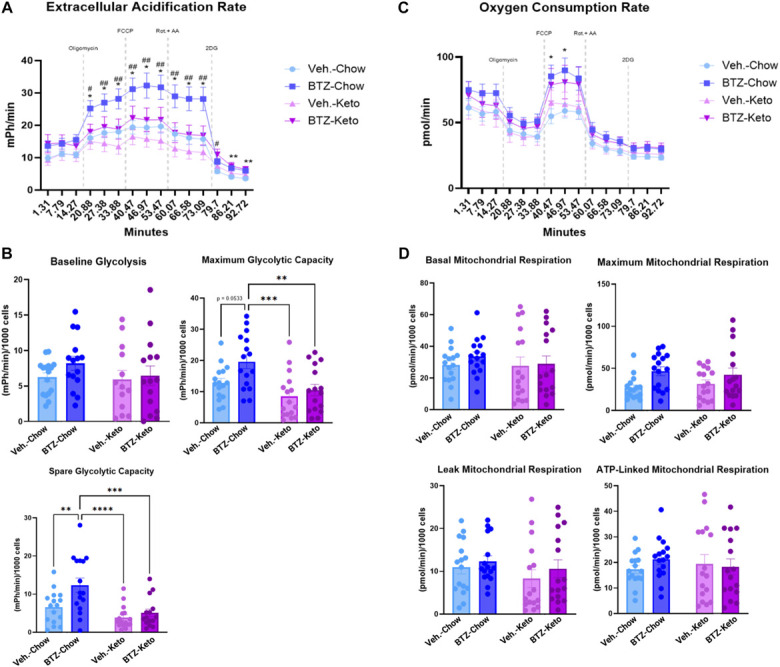
Results from metabolic assays performed on DRG neurons plated for 1 night in vitro, n = 4 mice, 4 to 8 replicates. Overall, extracellular acidification rate (*P < 0.05 BTZ-Chow vs Veh.-Chow; #*P* < 0.05 BTZ-Chow vs Veh.-Keto) (A) is depicted as well as specific glycolytic measures (B). Oxygen Consumption Rate (*P < 0.05 BTZ-Chow vs Veh.-Chow) was also measured (C) and is shown along with specific mitochondrial respiration measures (D). ATP, Adenosine Triphophate; BTZ, bortezomib; DRG, dorsal root ganglia.

Previous studies have shown that BTZ increases glycolysis in neurons.^34^ We measured the ECAR and OCR of DRG neurons after in vivo administration of drugs and diets. Results showed that baseline ECAR for all groups was not significantly different (Fig. 4A, 2-way ANOVA with repeated measures, group: *P* = 0.0085, group × time: *P* < 0.0001, time: *P* < 0.0001). However, BTZ-treated chow-fed animals had a significant increase in their DRG neurons' maximum glycolytic capacity (Fig. 4B, 2-way ANOVA, diet: *P* = 0.0005, drug: *P* = 0.0194) and spare glycolytic capacity when compared with all other groups (Fig. 4B, 2-way ANOVA, diet: *P* = 0.0002, drug: *P* = 0.0069). There were no significant differences for baseline mitochondrial respiration during OCR measure (Fig. 4C, 2-way ANOVA with repeated measures, *P* < 0.05), but there was a significant difference between BTZ-treated chow-fed animals compared with vehicle-treated chow-fed animals after FCCP injection (Fig. 4C, 2-way ANOVA with repeated measures, time: *P* < 0.0001). When looking at specific mitochondrial respiration measures, there were no significant differences between any groups (Fig. 4D, 2-way ANOVA, *P* < 0.05).

### 3.5. Ketones protect against bortezomib-induced neurite degeneration

**Figure 5.**
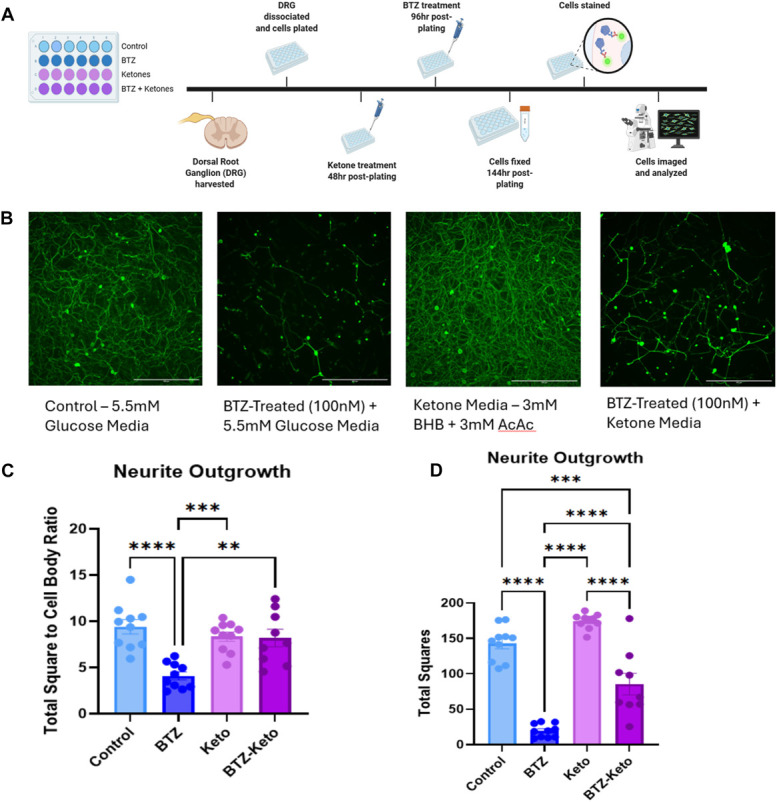
Illustration of the timeline (A) of in vitro study, n = 4 mice, 3 replicates. Scale bars = 500 µm. (B) shows representative photographs of lumbar DRG neurites. (C and D) Results of total square to cell body ratio (C) and total squares (D). BTZ, bortezomib; DRG, dorsal root ganglia.

We conducted a short-term course to identify whether exogenously provided ketones could prevent BTZ-induced neurite degeneration in vitro (Fig. 5A, B). Results showed that BTZ-treated DRG neurons exhibited a significant decrease in total square to cell body ratio (Fig. 5C, one-way ANOVA, group: *P* < 0.0001). In addition, when examining total squares, the ketone-treated group showed a significantly higher value than all other groups, including control cells (Fig. 5D, one-way ANOVA, group: *P* < 0.0001). Finally, the BTZ–ketone-treated group was significantly greater than the BTZ-treated group but remained significantly lower than the control and ketone-treated groups (Fig. 5D; 1-way ANOVA, group: *P* < 0.0001).

## 4. Discussion

Chemotherapy-Induced Peripheral Neuropathy (CIPN) is a substantial barrier when treating patients with cancer with chemotherapy drugs, and new treatment avenues are needed to lessen the neuropathy side effects induced by chemotherapy agents. Bortezomib is an excellent example, as the treatment of multiple myeloma using BTZ is strongly affected by BIPN. Here, we demonstrated that KD and ketones can prevent the development of BIPN symptoms, IENF loss, and neurite degeneration in a short-term, rapid BIPN development course. We also confirm that BTZ increases glycolysis,^34^ which may be linked to the development of nociception and pain symptomology observed in BIPN. Our data suggest that KD consumption prevents this increase in glycolysis, indicating that this bioenergetic change plays a role in neuronal activation in neuropathy. These bioenergetic corrections induced in sensory neurons by a KD add to the growing understanding that neuronal metabolism plays a central role in neuropathy. Another study conducted in a rat model of paclitaxel-induced peripheral neuropathy showed that PPARγ, which is important for lipid metabolism, is implicated in the mechanism underlying the benefits of ketones.^49^ Their work also highlighted that a KD reduced proinflammatory cytokine expression within the TLR4-NFκB pathway and significantly enriched oxidative phosphorylation pathways among differently expressed genes. This study aligns with our view that metabolism plays a key role in the benefits of a KD for neurons, and these results add to the growing body of evidence for the range of neuropathic conditions corrected by a ketogenic diet.

### 4.1. Ketogenic diet is well-tolerated by animals and induces ketosis

The KD is sustainable and maintainable, but the logistical burden and the diet's palatability can be onerous for some individuals.^3^ Our analysis suggests that the mice tolerated the KD, as BTZ-treated KD-fed mice weighed significantly less than vehicle-treated KD-fed mice; all mice remained within a healthy weight range throughout the study. Both KD-fed groups had a significant increase in blood ketone levels, showing that they had entered a state of ketosis.

### 4.2. Ketogenic diet prevents bortezomib-induced mechanical and cold hypersensitivity

Bortezomib treatment is known to cause nociception in animals^7,14,45^ and painful BIPN in humans.^47^ Previous studies have shown that BTZ-treated animals do not typically develop thermal sensitivity but instead develop cold sensitivity,^7,14^ leading us to measure cold sensitivity as well. Here, BTZ induced mechanical hypersensitivity in chow-fed animals, which persisted throughout the study; however, it did not affect KD-fed mice. Interestingly, BTZ induced cold sensitivity in both chow-fed and KD-fed animals at varying degrees. Ketogenic diet–fed animals did recover from cold sensitivity during the washout period, whereas chow-fed mice's sensitivity continued to climb. It is plausible that KD prevented mechanical allodynia but only mitigated cold sensitivity, given the different fiber types and mechanisms at play. These differences among neuronal phenotypes should be further explored.

### 4.3. Bortezomib causes fiber loss, but a ketogenic diet protects against fiber loss

It has previously been shown that BTZ reduces IENFD levels.^6^ Previous studies in our laboratory have shown that a KD can prevent reductions in IENFD in a mouse model of DPN. Here, our results showed that BTZ induces significant fiber loss within a short period of time, consistent with our previous studies on type 1 diabetes, type 2 diabetes, and high-fat diet-induced IENFD reductions.^10,17^ Importantly, BTZ-induced fiber loss can be prevented by consuming a KD. In addition, KD-fed animals have a heightened ability to grow neurites when harvested from KD-fed mice and placed in vitro.^10^ Although the mechanism is unknown, this strongly suggests that a KD can have lasting effects through improvements in metabolic memory, and experiments need to address the mechanisms underlying metabolic memory and how long they persist. This will be an essential feature for clinical translational use of a KD for CIPN.

### 4.4. Ketogenic diet inhibits bortezomib-induced metabolic changes within dorsal root ganglia neurons

This study in C57BL/6 mice showed that BTZ administration increased the ECAR in DRG neurons. These results are consistent with a similar study using BTZ to induce pain in ICR mice that first reported glycolytic changes.^34^ Our results strongly support this previous finding, showing that BTZ significantly increased the maximum and spare glycolytic capacities in DRG neurons compared with all other groups. This suggests that BTZ may alter the energetic phenotype of neurons toward a glycolytic state, which could contribute to the nociception observed in mice after BTZ administration. There is further evidence suggesting that unregulated glycolysis can lead to aberrant neuronal functioning and “glycolytic pain,”^22,28,31^ and that inhibiting excess glycolysis can reduce various pain phenotypes.^23,32,39^ Our results reveal that the BTZ-induced increase in glycolysis can be prevented by a KD, suggesting that one consequence of a KD is to modulate bioenergetics to prevent increases in glycolysis. This is consistent with the view that ketones can be directly used by neuronal mitochondria, bypassing the need for pyruvate-generated adenyl cyclase from glycolysis.^22,33,35^ Our results did not show changes in OCR in response to ketones or BTZ. However, the inhibition of glycolysis by ketones may be a beneficial mechanism, as acetoacetate has been shown to inhibit glucose uptake and downstream glycolytic events.^37^ Moreover, glutamine added to the media can be metabolized by mitochondria, potentially masking differences in OCR. Future studies should test how glutamine in the media affects neuronal bioenergetics. In addition, our cultures were not pure neurons, and future studies should investigate the impact of non-neuronal cells. Finally, we recognize that in vivo elevations in ketones may have led to metabolic adaptations in cells in vitro, thereby affecting the results. Overall, this relationship between glycolysis and mitochondrial oxidative phosphorylation merits further study, as it seems to play a crucial role in sensory neuron function, neuropathy, and pain.^22,33,35^

### 4.5. Ketones promote growth and prevent bortezomib-induced neurite degeneration

It is known that BTZ causes neurite degeneration in vitro,^42^ but there is little research surrounding preventing BTZ-induced neurite degeneration. Previous work from our laboratory has demonstrated that ketones can promote neurite outgrowth in vitro.^10^ Here, the addition of ketones in vitro 2 days before BTZ significantly mitigated neurite loss, supporting the effect on IENFD in vivo and suggesting that ketones may protect neurites by altering their bioenergetics.

## 5. Limitations

We focused on a short time course to identify alterations in sensory behaviors, IENFD, and cellular bioenergetics. This paradigm does not reflect the duration of time patients experience when receiving BTZ. Future studies should expand the time course to identify changes in nociception and cellular bioenergetics, and examine repeated BTZ dosing regimens or exogenous ketones to translate these effects better. In addition, a KD administered to rodents is believed to be protein-deficient, which may result in increased signaling pathways related to protein deficiency that act independently of ketone-mediated effects.^29^ Further studies could focus on ketone body metabolism and the impact of inhibition on it. Finally, as a starting point, we only conducted these studies in male mice. There seems to be no sex difference in the response of humans or rodents to BTZ treatment,^5,11^ and additional studies in female mice may be warranted. Recent work has shown that there are sex differences in KD efficacy for weight loss in humans, concluding that these differences may be attributed to factors such as sex hormones, neurotransmitters, genetics, immunity, and gut microbiota.^25^ Finally, to avoid contraindications, patients should consult their doctors before starting a KD to ensure proper education and maintenance.

## 6. Conclusion

We report that BTZ can induce rapid distal axon degeneration, and that a KD can prevent reductions in IENFD, mechanical allodynia, and a neuronal glycolytic shift in BTZ-treated mice. In addition, ketones can prevent BTZ-induced neurite degeneration in vitro. Our study underscores the benefits of a KD in a BIPN model and highlights bioenergetic changes that may provide benefits in neuropathy. Future studies could focus on a rescue intervention for BIPN with KD, or on a more intensive prevention paradigm in which KD is administered earlier. Finally, further exploration regarding the metabolic alterations occurring with DRG neurons as a result of drug and diet administration should be pursued.

## Disclosures

The authors have no conflicts of interest to declare.
